# Envisioning a Multidisciplinary HBV Cure Research Agenda

**DOI:** 10.1007/s11904-025-00763-y

**Published:** 2025-11-08

**Authors:** Karine Dubé, Ali Ahmed, Chari Cohen, Yasmin Ibrahim, George A. Yendewa, Edward R. Cachay, Su Wang, Kristen Marks, Arthur Y. Kim, Jeremy Sugarman, David L. Thomas, Chloe L. Thio, Debika Bhattacharya

**Affiliations:** 1 Division of Infectious Diseases and Global Public Health, University of California San Diego School of Medicine, 9500 Gilman Drive, 0507 La Jolla, CA USA; 2 Hepatitis B Foundation, Doylestown, PA USA; 3 Case Western Reserve University School of Medicine, Cleveland, OH USA; 4 Cooperman Banabas Medical Center, RWJ Barnabas-Rutgers Medical Group, Livingston, NJ USA; 5 Weill Cornell Medicine, New York, NY USA; 6 Harvard Medical School, Boston, MA USA; 7 Johns Hopkins Berman Institute for Bioethics, Baltimore, MD USA; 8 Johns Hopkins University School of Medicine, Baltimore, MD USA; 9 David Geffen School of Medicine, University of California Los Angeles, Los Angeles, CA USA

**Keywords:** Socio-behavioral sciences, Ethics, Community engagement, HBV cure research, HBV-HIV co-infection, Review

## Abstract

**Purpose of Review:**

We examine the current understanding of the multidisciplinary aspects of hepatitis B cure research, such as socio-behavioral sciences, ethics, community engagement, and translational and implementation science.

**Recent Findings:**

The peer-reviewed literature on the multi-disciplinary aspects of HBV cure research is gradually expanding, although several areas still require attention. These deficiencies include: the acceptability of HBV treatment discontinuations, HBV-related stigma, the impact of co-infections (e.g., HIV), and the translation of discoveries to resource-limited settings.

**Summary:**

This review highlights the importance of a multidisciplinary framework that bridges socio-behavioral sciences, ethics, community engagement, and translational and implementation science to help ensure the development of an effective, acceptable, scalable and equitable HBV cure.

## Introduction

Hepatitis B virus (HBV) is a major global health threat, with nearly 300 million people worldwide living with chronic hepatitis B (CHB) [1, 2]. Despite the availability of effective vaccines and antiviral treatments, CHB continues to cause significant burden, leading to around 1 million deaths annually from cirrhosis, liver failure, and liver cancer [1, 3]. About 10% of people living with HIV (PLWH) also have CHB [4, 5].

Treatment with nucleos(t)ide analogues (NAs), the main therapies for CHB, often leads to HBV deoxyribunucleic acid (DNA) becoming undetectable and liver inflammation improving. However, despite these therapies, the template for replication, the covalently closed circular DNA (cccDNA), and HBV integrated into the host genome (integrated HBV DNA) remain, along with secreted hepatitis B surface antigen (HBsAg) [6, 7]. This poses a risk of HBV reactivation with discontinuation of therapy or during immunosuppression [8]. Given the global morbidity and mortality associated with HBV, the challenges of long-term NA treatment, and the stigma surrounding the disease [9–12], considerable efforts are being taken to develop a cure for CHB. There is growing interest in curing CHB from a wide range of stakeholders, including the U.S. National Institutes of Health (NIH), industry, academia, and the patient and advocacy community, with over 40 compounds currently in various stages of development [12–14].

HBV cure can be classified as either a *functional cure* (sustainable loss of HBsAg and undetectable plasma HBV DNA, after finite treatment) or a *complete cure* (elimination of all viral replicative particles, including cccDNA) and harboring cells). Functional cure is scientifically plausible, as it occurs spontaneously in 90% of adults without immunosuppression after acute HBV infection [4, 15–17]. However, achieving a complete cure is more difficult, as current therapies and spontaneous resolution do not eliminate cccDNA or integrated HBV DNA, which are responsible for antigen expression, including HBsAg. While people with both HBV and HIV are at higher risk for developing CHB, cirrhosis, end-stage liver disease (ESLD), and hepatocellular carcinoma (HCC) [4, 18, 19], they are paradoxically more likely to achieve functional cure following the initiation of HIV antiretroviral treatment (ART) that both targets HBV and restores immunity [15]. However, stopping treatment to test the efficacy of a potential HBV cure presents significant challenges, as discontinuation may cause HBV to reactivate, potentially leading to serious flares of hepatitis [15, 20].

Several recent advances in novel antiviral agents, virologic and immune modulation strategies, as well as gene-editing techniques offer promising prospects for HBV cure [21–23]. However, HBV cure research has broad implications extending beyond scientific and clinical matters and include important socio-behavioral and ethical issues. Akin to the recognized importance of behavioral and social sciences research (BSSR) to HIV cure research [24, 25], we propose the creation of a similar framework for HBV cure research.

**Fig. 1:**
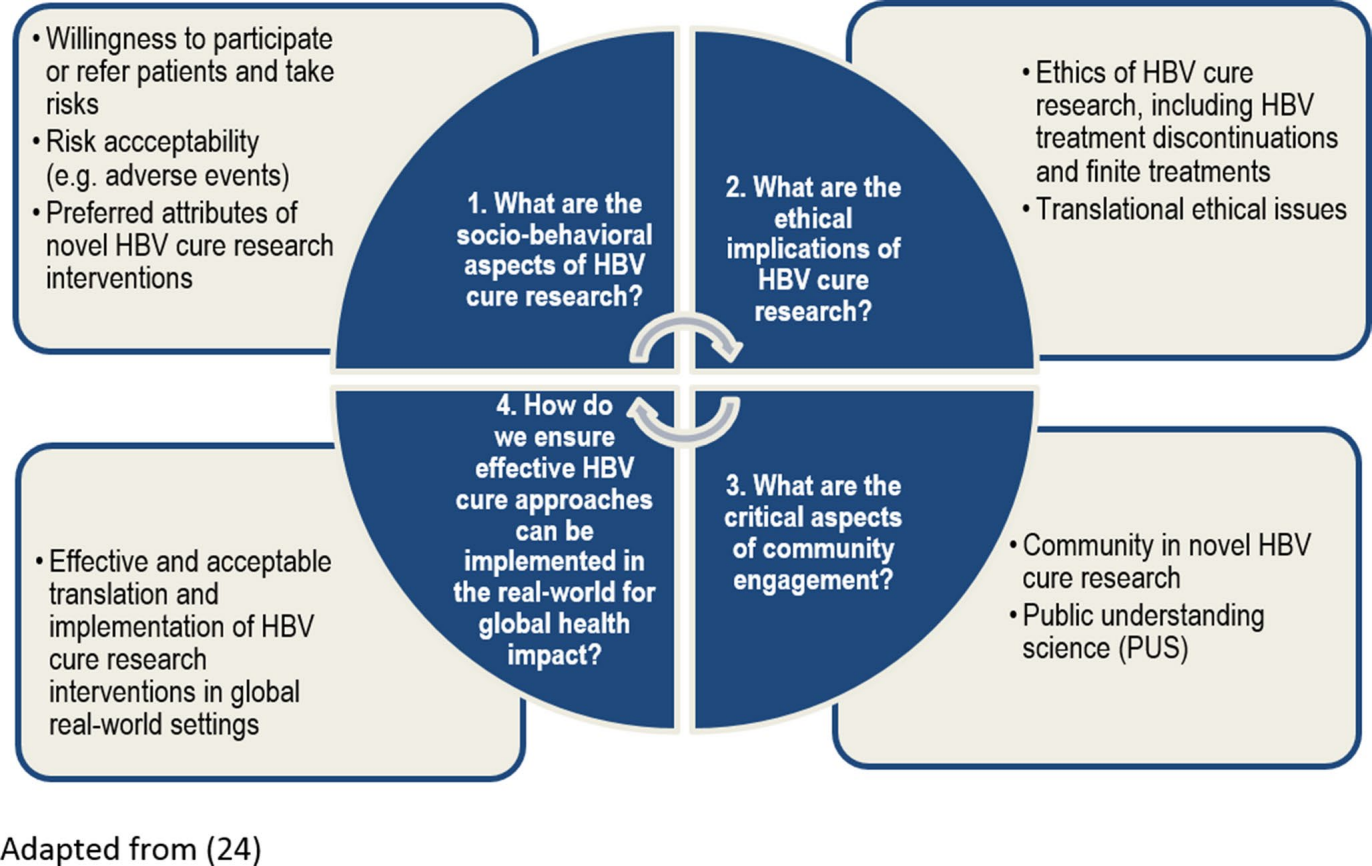
Conceptual Framework – Envisioning a Multidisciplinary HBV Cure Research Agenda. Adapted from [24]

Our proposed multidisciplinary approach adapts a framework (Fig. 1) from HIV cure research [24] to HBV cure research. This approach integrates four domains to basic and biomedical research: (1) socio-behavioral sciences; (2) ethics; (3) community engagement; and (4) translational and implementation science. Adapting this approach for HBV cure research recognizes key similarities between both viruses, including their integration into host DNA (albeit non-replicating HBV DNA vs. replication-competent HIV provirus), latency in long-lived cell populations (hepatocytes vs. CD4 + T cells), and the clinical and psychosocial risks involved in developing novel therapies. While HCV cure research also provides valuable insights, complete HCV cure has been achieved but remains aspirational for HBV and HIV. As such, the HIV cure research framework provides a relevant model for guiding a multidisciplinary HBV cure research agenda [26, 27].

Envisioning a multidisciplinary approach to HBV cure research is crucial for advancing the field. Socio-behavioral research provides insights into how affected communities perceive HBV, engage with novel therapies, and respond to the potential of a cure, while addressing factors such as stigma and the psychosocial impacts of research. Ethics involves determining what ought to be done, ensuring among other issues that trial designs and conduct, including treatment discontinuations, are ethically and scientifically appropriate [28]. Community engagement comprises meaningfully involving affected populations throughout all stages of research, fostering trust and promoting equity. Translational and implementation science facilitates the translation of promising discoveries into real-world applications. Together, this multidisciplinary approach helps ensure that HBV cure research is scientifically robust, ethically sound, and socially relevant.

## Methodology

### Conceptual Framework

This review adapts a conceptual framework for multidisciplinary HIV cure research [24] to organize socio-behavioral, ethics, community engagement, translational and implementation science priorities relevant to HBV cure research.

### Review Methods

From December 2024 – October 2025, we reviewed journal articles published in English related to the four domains outlined above. Given the limited literature available, we did not restrict our selection by publication date and included various article types, such as original research articles, reviews, and viewpoints. We initially searched PubMed, using search terms such as “social sciences”, “ethics”, “engagement”, “translation” and “implementation” AND “hepatitis B cure research.” We then screened titles, reviewed abstracts, and conducted full article evaluations. We used a snowball sampling approach to identify additional relevant sources. We also integrated recommendations from sources outside of HBV cure research that could offer insights, such as examples from HIV or HCV cure research. We identified key themes and priorities through iterative review of the literature and expert input from the co-authors.

For each domain, we provided an overview, summarized existing knowledge, and identified potential knowledge gaps.

## Review of the Literature

### Socio-Behavioral Sciences of HBV Cure Research

#### Overview

A socio-behavioral research lens promises to enhance the HBV cure research agenda by addressing the social, behavioral, and psychological factors that shape people’s understanding, acceptance, and adherence to future HBV cure trials and interventions [25, 29].

#### What is Known

There has been limited research on the socio-behavioral aspects of HBV cure research. A systematic review of articles published between 1980 and 2024 [30] found only two peer-reviewed articles on attitudes of persons living with hepatitis B (PLWHB) toward novel HBV therapeutics, highlighting the need for further research into attitudes, willingness to participate, and perspectives on HBV cure among PLWHB.

The first study, conducted in Germany between 2018 and 2019, used a discrete choice experiment to assess the preferences of 108 people with CHB without a history of HCC or HIV/HBV co-infection regarding a functional cure [31]. The results revealed that efficacy, particularly the potential for sustained treatment-free HBV control, was the most important factor affecting acceptability, influencing 57% of participants’ decisions. Other factors included the treatment regimen (17%), safety profile (12%), and frequency of physician visits (11%). Participants preferred oral administration over subcutaneous injection or electroporation and favored fewer physician visits and minimal side effects. The second study [32] involved interviews conducted with 19 people with CHB across the United States, most of whom were taking HBV antiviral treatment. Key themes on the acceptability of HBV cure research included potential benefits such as the ability to stop medication, improved energy levels, and reduced anxiety about lifelong infection and liver cancer risk. However, participants also expressed concerns about potential side effects, frequency of administration, duration, cost-effectiveness, and overall impact on quality of life, and wanted to see clinical research data that reflected their demographics [32]. The geographic and cultural scope of the two above studies [31, 32], however, limits their generalizability to under-resourced settings or more diverse populations.

In addition, a report by the Hepatitis B Foundation gathered responses from over 2,000 participants across 102 countries about their ideal outcomes for novel HBV control regimens [33]. The most common preferences included loss of HBsAg (28%), stopping medication after 6–12 months (24%), and reduced liver cancer risk (20%). Additional desired outcomes included improved quality of life (14%), loss of HBV cccDNA (9%), and sustained undetectable HBV DNA (5%). Accounts of what it means to achieve a hepatitis cure can also provide valuable insights into desired cure outcomes. Richmond and colleagues [34] documented the experiences of 20 people formerly living with HCV, highlighting improvements in psychological well-being, reduced fear of developing liver disease or cancer, and decreased concern about transmitting HCV.

The extant literature on HBV treatment experiences [35] and lived experiences and unmet needs of PLWHB also offer important information [36–41]. Published reports highlight the significant psychosocial impacts of HBV, such as reduced quality of life [42, 43], stigma [9–12], discrimination [44], fear [45], anxiety [37, 45], and financial instability [46], which are not alleviated by current HBV therapies. These challenges are further compounded in populations that live with HBV and other co-infections, such as HIV. Research on the lived experiences of PLWHB [12, 45, 47] underscores the psychological toll of CHB, revealing issues such as guilt, social withdrawal, and ambiguous prognoses. These findings suggest that the adoption of a journey approach to HBV cure research, considering how people live with and experience novel therapies, should be of value.

#### Gaps in Knowledge

As the landscape of HBV therapeutics evolves with promising long-acting treatments [48], there is a concomitant need for research on the acceptability of novel HBV therapeutic approaches. While clinical safety and efficacy remain central to the development of these therapeutics, patient acceptability is a significant factor influencing treatment and cure outcomes [30–32, 49]. One major gap in knowledge is identifying correlates of acceptability [50], which encompass demographic, psychological, cultural, and socio-economic factors. These factors are essential for aligning product development with patient’s preferences [50]. For instance, some PLWHB may prioritize approaches that allow them to maintain their usual lifestyle, while others may prioritize efficacy over convenience [30].

The acceptability of what constitutes efficacy is critical [26, 51, 52]. PLWHB may define an ideal treatment outcome, or “cure” differently. For some, not having to take medication indefinitely would be an important goal; for others, not needing to take medications would not be enough if there remained a risk of future recurrence. Others might focus on eliminating the risk of transmitting infection to others. Understanding these preferences matters since the medical strategies for achieving each might differ. Additionally, PLWHB’s tolerance for side effects compared to traditional treatments remains a critical knowledge gap [52]. It has been argued that PLWHB want affordable finite treatment with strong safety profiles that will reduce their risk of liver cancer and lead to HBsAg loss [32]. Yet to date, there are no published studies describing the perspectives of PLWHB on partial cure (finite treatment, sustained off-treatment HBV DNA suppression, no HBsAg loss), which will be helpful to understand to make decisions for investing in such research.

Another important area is acceptability of intervention types and attributes [51]. This includes routes of administration, frequency of administration, convenience, and potential side effects, pain or discomfort. Further, the acceptability of invasive procedures, such as liver biopsies or fine-needle aspirations, remains understudied, despite their necessity for quantifying cccDNA and the hepatic reservoir. To date, a single study has explored the acceptability of liver biopsies in the context of hepatitis care. Amorosa and colleagues [53] examined the willingness of 235 people with HCV, including 113 also living with HIV, to undergo repeat liver biopsies. The study found that 86% of participants were willing to repeat the procedure and highlighted the importance of informing patients about the liver biopsy’s utility and safety.

Another gap in understanding is the acceptability of HBV treatment discontinuations [30, 54] (discussed further below). The willingness of PLWHB to discontinue treatment may be influenced by their perceptions of risk or disease progression. Moreover, for people with co-infections such as HIV, who need to take medications for their HIV, the benefit of eliminating indefinite HBV treatment might be less than for someone for whom that is the only medication.

Patient-reported outcomes (PROs) [55], including the lived experiences of trial participants [6, 56], can provide important insights into the broader dimensions of HBV cure research acceptability. These outcomes, encompassing mental health impacts, social stigma, and quality of life, can inform both clinical practice and the development of future HBV therapeutics. Studies have assessed the development of PROs for hepatitis B [57, 58]. There is at least one psychometrically sound quality of life instrument that captures the multi-faceted constructs associated with CHB (i.e., physical, emotional, and social, including stigma) [57]. Beyond direct health gains, the societal and psychological value of a cure [59], such as increased social inclusion or reduced anxiety, should be explored to ensure a holistic approach to research. Experiences from the HIV field illustrate the value of incorporating patient-centered outcomes into clinical and research agendas. For example, adoption of the HIV360 outcome set has enabled healthcare providers to record, compare, and integrate standardized metrics across treatment sites, thereby driving quality improvement in HIV care and ensuring that outcomes reflect what matters most to people living with HIV [60]. This shift toward standardized, patient-reported outcomes underscores the importance of defining “value” in terms that extend beyond clinical markers. Establishing a similar, harmonized outcome framework for hepatitis B could facilitate the integration of biomedical and psychosocial dimensions of care, advancing both cure research and long-term well-being for PLWHB. Understanding these multifaceted elements of acceptability will be critical for creating HBV therapeutics that are not only clinically effective but also aligned with what matters most to PLWHB.

Corneli and colleagues [61] outlined five approaches for integrating behavioral and social sciences into clinical trials in general (formative, embedded, parallel, explanatory, and implications), each of which is categorized by timing (before, during, or after the trial). To achieve this, a variety of research methods, including qualitative, quantitative, mixed methods, and conjoint analyses, will be necessary. Additionally, while PROs are important to PLWHB, inclusion of PRO assessments in HBV clinical trials and clinical management will require a shift in the clinical current clinical approach, which uses only biomarkers and does not include quality of life to evaluate both disease impact and treatment response.

Briefly, a socio-behavioral research lens promises to enhance the HBV cure research agenda by addressing psychosocial, cultural, and contextual factors that influence intervention adherence, implementation, and equitable access, ensuring therapies are tailored to the needs of diverse affected populations.

## Ethics of HBV Cure Research

### Overview

Normative and empirical ethics research can contribute to the HBV cure research agenda by addressing critical ethical considerations in the development, testing, and implementation of HBV cure research interventions.

### What is Known

Sugarman led two critical reviews on the ethics of HBV cure research. The first seminal review [6], published in *Gut*, outlined five key ethical considerations for the field. These include: (1) minimizing risks of interventions, including ensuring proper clinical monitoring; (2) selecting appropriate trial outcome measures [52]; (3) identifying proper study populations, which may include underrepresented groups such as migrant populations and individuals who use drugs and disproportionally affected by HBV [62]; (4) ensuring participants provide informed consent and fully understand the nature of the research, especially regarding the use of the term “cure”; and (5) promoting fairness, such as conducting research across different HBV genotypes.

The second review [27], published in *Current Opinion in HIV/AIDS*, compared ethical issues in HBV and HIV cure research. This review highlighted significant gaps in the ethics of HBV cure research, including limited stakeholder engagement, insufficient assessment of HBV-related stigma, and a general lack of understanding of participants’ perceptions and attitudes toward ethical HBV cure research. The review emphasized the importance of these components in informing ethical deliberations and called for increased scholarly attention to strengthen the ethical foundations of HBV cure clinical research.

### Gaps in Knowledge

Additional gaps will need to be addressed to ensure that HBV cure research is conducted ethically and equitably for PLWHB, as well as those with co-infections. Like HIV cure research that involves analytical treatment interruptions (ATIs) [63–65], HBV treatment discontinuations and finite treatments pose significant challenges. To move the field of HBV cure research forward, it will be imperative to develop ethical recommendations around HBV treatment discontinuations and finite treatments [54, 66–69]. This guidance will need to specify the inclusion and exclusion criteria for trials, monitoring strategies and measures, and treatment restart criteria. Understanding patients’, providers’ [70], researchers’ and regulators’ perspectives on HBV treatment discontinuations will be critical to advancing HBV cure research. Special considerations should also be given to resource-limited settings regarding treatment discontinuations, as monitoring may be more challenging due to limited access to HBV DNA and alanine aminotransferase (ALT) testing, often driven by cost constraints, raising important ethical considerations.

Additional ethical issues in HBV cure research include considerations of informed consent and supportive decision-making [71, 72], HBV cure intervention-specific considerations [73, 74], mitigating third-party risks during HBV treatment cessations, such as the need for sex and drug use partners to have been vaccinated for HBV, while ensuring adequate privacy protections [6, 75]. Notably, there is a lack of guidance on how researchers should balance protecting third parties with safeguarding participants’ privacy rights. Other concerns include ethics of trial designs, including the use of placebos, and special considerations for different sex or gender and for pediatric HBV cure trials. A knowledge gap remains concerning the ethical considerations associated with adaptive trial designs, multi-arm studies, and trials involving potentially vulnerable populations, such as children or pregnant individuals.

HBV cure research will also need to also be guided by equity [76], human rights [77] and justice-informed paradigms [78] to ensure fair access to research opportunities and future interventions, particularly for underrepresented populations and those in resource-limited settings. Currently, there is a high willingness among PLWHB to participate in clinical trials globally, but access is limited in many countries [29, 79], including those most highly impacted. HBV clinical trials need more patient-centered designs. Additionally, the development of an HBV cure may unintentionally exacerbate stigma, particularly for people with both HIV and HBV facing dual stigma, necessitating proactive efforts to minimize social harm. Stigma must be recognized as a significant ethical concern, and efforts should be made to quantify it, understand its roots and direction, and develop tailored interventions, such as public education campaigns and destigmatizing language guidelines, to mitigate stigma both during research and post-cure implementation. Given the uncertainty surrounding the long-term safety and effectiveness of future HBV cure interventions, ongoing clinical monitoring will be necessary, along with ensuring accessible post-cure follow-up care. These requirements will necessitate considering burdens, costs and privacy.

Briefly, the ethics of HBV cure research will involve navigating complex issues such as ensuring the safety and well-being of those enrolled in HBV research, obtaining their meaningful informed consent, securing equitable access, reducing stigma, ensuring patient-centricity, and preventing unintended long-term consequences. Addressing these issues requires a comprehensive ethical framework to guide the pursuit of an HBV cure that is fair, inclusive, and aimed at benefiting the populations most in need. This framework should include ensuring transparency in the research process, such as reporting adverse events, sharing trial data, and involving community members in oversight roles.

## Community Engagement in HBV Cure Research

### Overview

Community engagement is essential to advancing HBV cure research by ensuring inclusivity, equity, cultural relevance, and fostering collaboration between researchers, care providers, and affected communities.

### What is Known

Hepatitis B affects specific global communities, with the highest prevalence in Asia, Africa, the Western Pacific, and the Eastern Mediterranean [1]. In the U.S., HBV disproportionately affects Asian American, Pacific Islander, and African communities, underscoring the need for tailored engagement strategies, particularly for populations with limited English proficiency [80–84].

In 2023, the declaration of PLWHB called for a whole-person approach to care and research, recognizing the full impact of the disease on people’s lives [13]. In 2025, Lazarus and colleagues published the People-First Liver Charter, advocating for person-first language [85].

Borondy-Jenkins and colleagues [86] reported on lessons learned from establishing an inaugural global Hepatitis B and D community advisory board (CAB), convened by the Hepatitis B Foundation in 2022. The CAB, which included 23 members from 17 countries, represented regions with the highest HBV and HDV prevalence. Hepatitis delta (HDV), a common co-infection with HBV, affects between 5 and 10% of PLWHB [86]. The report highlighted participants’ motivations to advocate for people with HBV and HDV. While CAB members gained valuable networking and advocacy opportunities, they also faced challenges such as time commitments, stigma, and difficulties engaging with novel HBV drug developers.

Careful considerations must be given to the engagement for HBV cure trials. It is important to distinguish between engagement, which involves ongoing dialogue, and recruitment, which focuses on identifying eligible participants [87]. Engagement should be an ongoing process, extending beyond recruitment and trial participation, and aligned with global equity goals. It is also essential to view engagement as a multi-stage process, encompassing all stages of research from defining questions to disseminating findings and implementing interventions [88].

Cornberg and colleagues [52] describe a framework for prioritizing populations in HBV cure trials, recommending that initial focus be on people with the greatest need and potential benefit from effective regimens, while also considering other groups. Their guidance aligns with the U.S. Food and Drug Administration (FDA) mandate to include diverse populations in clinical trials, particularly regarding race and ethnicity [89].

The emphasis on inclusivity aligns with the concerns raised by Mofokeng and colleagues [90] who stressed the need for greater awareness and information to improve participation in HBV clinical research, particularly in resource-limited settings like South Africa. They identified trial participation as a key barrier, alongside other challenges such as lack of linkages with research teams that hinder effective engagement [90]. Similarly, a study by Zovich and colleagues [91] provided insights into effective communication strategies for engaging affected communities. Their findings emphasized the importance of culturally appropriate approaches and found that communities prefer materials in both English and native languages while highlighting the importance of avoiding stigmatizing language.

### Gaps in Knowledge

There are several gaps in knowledge regarding effective community engagement in HBV cure research, primarily due to the scarcity of HBV advocacy programs compared to HIV [92]. Effective participatory practices in HBV cure research are also lacking, particularly in clinical research settings where the voices of people with lived experiences must be integrated into the design of clinical trials [93]. Further, there is a need to engage communities in defining appropriate terminology relevant to HBV cure research, ensuring that complex terms like “functional cure” are clearly understood and non-stigmatizing [86, 94]. It is also essential to explore how communities perceive and weigh various notions of “cure” (e.g., complete, partial, durable, absolute, immune, remission, finite), including the different antibody expressions linked to these terms [52]. In HIV cure research, expressions such as “sterilizing cure” [95], “subject”, and “infected” when used in reference to people can be considered stigmatizing and should be avoided while the use of person-first language is highly encouraged.

To bridge the gap between PLWHB and biomedical researchers, CABs will be essential for empowering community members and ensuring their active involvement in HBV cure research. Further, enhanced training on HBV cure research, the establishment of an HBV/HIV Community Advisory Board for guidance on co-infection issues, and innovations in health communication to simplify complex scientific concepts could help strengthen these efforts. Improving public understanding of science [96], assessing meaningful social [97] and community engagement [98], and documenting lessons learned from equitable community engagement models should facilitate the success of HBV cure research [87, 99]. Additionally, there is need for capacity-building programs for local researchers and communities, development of patient-centered engagement metrics, and strategies for engaging under-represented groups, such as rural populations and those with lower health literacy levels. Adaptive strategies that account for cultural and gender-specific factors in diverse settings will ensure that all communities are meaningfully involved in the research process. Finally, developing trust-building strategies promise to foster strong, sustained partnerships with communities, ensuring that HBV cure research is inclusive, transparent, and ethically grounded [100].

Briefly, incorporating community engagement in HBV cure research is crucial for developing culturally relevant, equitable, and inclusive interventions that address the unique needs of affected communities, build trust, increase participation, and facilitate the downstream implementation of HBV cure strategies.

## Translational and Implementation Science

### Overview

Translational and implementation science can help bridge the gap between HBV cure research and its eventual real-world applications. Translational science focuses on moving promising therapies from the bench to clinical practice. However, effective national and global health practices require flexible, effective, and scalable models, particularly in resource-limited settings most affected by HBV. The unique challenges faced in these regions, such as healthcare infrastructure constraints and limited diagnostic capacity, require tailoring implementation strategies and promoting equitable policies. It is also essential to anticipate the development and distribution of generic medications that can be integrated to overcome cost barriers.

### What is Known

There has been limited research on the implementation of HBV cure interventions. An article by Wallace et al. [101] examined the public health and social implications of implementing future HBV cure interventions. Based on 31 interviews with professional stakeholders, the study identified key factors for successful HBV cure access, including health system preparedness, the need for healthcare infrastructure and qualified professionals, and equitable resource allocation. The authors emphasized lessons learned from HCV cure implementation, such as proactive case finding and treatment linkage, while highlighting disparities in HBV prevention, treatment, and care. The article also highlighted important social implications of HBV cure, noting that desired cure outcomes, such as clearing HBsAg or achieving antibody-negative status, may differ across regions. The authors called for further research to better understand the social, cultural, and political factors involved in HBV cure implementation.

An article by Jackson et al. [102] explored barriers to accessing hepatitis B medications, identifying financial challenges, health insurance issues, and pharmacy preauthorization processes, as well as stigma and a lack of reliable, patient-friendly information. These barriers were found to significantly affect continuity of care for PLWHB, hindering their access to necessary treatments, let alone curative regimens.

### Gaps in Knowledge

Research on the translation and implementation of HBV cure faces significant knowledge gaps, particularly in anticipating issues across the entire research and implementation cascade. This includes challenges from basic and preclinical research to human clinical trials and eventual real-world applications. The Khoury T0–T4 continuum of translational research describes the process from basic discovery (T0), through pre-clinical (T1) and clinical testing (T2), to clinical implementation (T3) and eventual public health application (T4) [103]. The T4 stage, focused on improving population health, highlights scalability and maximizing translational social value [104], which can be informed by tools like target product profiles (TPPs), commonly used in drug development to align stakeholders on desired research outcomes [105, 106]. Actionable strategies to enhance scalability and translational potential include partnering with governments and non-governmental organizations to subsidize therapies, utilizing point-of-care testing to improve diagnosis in remote areas, and leveraging community health workers to enhance the delivery of HBV treatments. While the Khoury T0 – T4 continuum of translational research framework [103] has been applied in HIV cure research [49], similar frameworks for HBV cure remain underexplored.

Further, cost-effectiveness, access (107), and affordability remain significant concerns, particularly considering the high initial costs seen with HCV cure implementation, which should not be considered a desirable model for HBV. Instead, successful global health interventions like HIV treatment scale-up may offer a better roadmap. Economic analyses of HBV cure strategies are limited but essential for policy decisions and addressing access disparities (108). Translational science must consider the economic impact of HBV cures and the long-term clinical and psychological challenges for survivors [49]. The HCV cure development experience highlights the importance of integrating access considerations early to prevent health inequalities (109). Effective implementation science and capacity-building, through improved infrastructure, staff training, and financing, will likely be needed for HBV cure adoption globally. Health financing mechanisms, such as pooled procurement and subsidies, should be addressed to make HBV cure strategies affordable. Co-infections of HBV/HIV and HBV/HDV, particularly in resource-limited settings, will require further research and implementation efforts.

Expanding access to hepatitis B vaccination remains an essential cornerstone for achieving global HBV elimination. Global initiatives such as Gavi, the Vaccine Alliance, have played a critical role in increasing vaccine coverage, particularly in low- and middle-income countries, thereby preventing new infections and reducing long-term disease burden (110). The experience of the U.S. President’s Emergency Plan for AIDS Relief (PEPFAR) offers additional lessons for the global HBV response. PEPFAR’s extraordinary impact on reducing HIV-related morbidity and mortality in sub-Saharan Africa demonstrates how coordinated, well-funded, and evidence-based implementation programs can transform the trajectory of an epidemic (111). A similar global initiative for HBV, focused on scaling up vaccination, testing, linkage to care, and antiviral treatment, could substantially advance elimination goals in highly endemic regions. Integrating implementation science into such efforts would further ensure that programs are locally relevant, sustainable, and centered on the needs of affected communities.

Monitoring and evaluation systems will be needed to track the long-term effectiveness of HBV cures in real clinical settings. These systems allow for continuous assessment of cure sustainability, adverse effects, and overall treatment success across different populations. Key questions remain: who will bear the financial burden, and what partnerships, such as those with the World Health Organization (WHO), the private sector, or other stakeholders, can be leveraged to support these efforts? Addressing these critical issues is essential for the successful global implementation of HBV cure strategies. By gathering real-world data, healthcare systems can identify barriers to effective implementation and adapt strategies accordingly. Ongoing evaluation ensures that HBV cure interventions remain equitable, meeting the needs of diverse underserved populations while adjusting to local contexts.

Briefly, foresight on translational and implementation issues will be important to ensure safe and effective HBV cure strategies are seamlessly integrated into healthcare systems. This lens can help accelerate the transition of discoveries from the laboratory to real clinical settings [23], ultimately making HBV cure more equitable, sustainable, and accessible to diverse global populations. The success of HBV cure implementation will depend on global collaboration, local adaptability, and equitable distribution systems. Achieving this will require the development of cost-effective therapies, support for generic manufacturing, and the integration of implementation science frameworks, all tailored to the realities of resource-limited settings. Only by aligning these efforts can we ensure that HBV cure strategies are not a privilege for the few but a transformative intervention that has the potential to improve the lives of many.

### Future Research Directions

Table 1 summarizes outstanding socio-behavioral, ethics, community, translational, and implementation research questions emerging from our review. Recurring themes and challenges, such as the acceptability of HBV treatment discontinuations, HBV-related stigma, and the impact of co-infections (e.g., HIV, HDV), intersect across multiple framework domains. This includes recognizing the rights of persons to access information, care, and interventions, while also encouraging responsibilities such as adherence to preventive measures and engagement in clinical research when appropriate. In contexts of limited resources, transparent prioritization of funding and research agendas will also be essential. Advancing an agenda for HBV cure will require the active engagement of all relevant stakeholders, such as health administrators, communities, and people at higher risk or already living with HBV.

**Table 1:** Outstanding Socio-Behavioral, Ethics, Community Engagement and Translational and I mplementation Research Questions towards Multidisciplinary HBV Cure Research

1. **Socio-Behavioral Issues**
• How do demographic factors influence patients’ willingness to accept novel HBV therapeutics and the perceived value of a “cure”?
• What psychological factors shape patients’ understanding and expectations of HBV cure strategies, particularly in relation to HBsAg loss versus complete elimination of cccDNA?
• How do cultural and social norms affect the acceptability of novel HBV clinical therapeutics in light of the evolving landscape of long-acting HBV therapies?
• What is the role of stigma, and dual or compounded stigma for people with co-infections (e.g., HIV, HDV), play in the willingness to participate in HBV cure trials or interventions?
• What factors influence patients’ acceptability of invasive procedures like liver biopsies or fine-needle aspirations?
• Under what conditions would PLWHB be willing to interrupt HBV treatment to advance HBV cure research?
• What are the unique challenges and considerations in the acceptability of HBV treatment discontinuation for people with co-infections like HIV or HDV?
• How can patient-reported outcomes (PROs) of PLWHB be effectively integrated into clinical trials and hepatitis B management to ensure that treatment strategies align with the needs and priorities of this population?
• What clinical outcomes are most meaningful to PLWHB, and how can these outcomes be incorporated into drug development processes, while balancing the interests of patients and drug developers, particularly in terms of ensuring both patient-centered care and commercial feasibility?
• How do patient-reported outcomes related to mental health and quality of life inform our understanding of the broader impact of HBV cure therapies beyond clinical health gains?
• How can standardized, person-centered outcome frameworks like HIV360 be adapted or developed to capture what matters most to PLWHB?
• What are the socio-cultural barriers and facilitators to equitable access to HBV cure interventions, and how can these be addressed to improve the uptake of new treatments among marginalized populations?
• In what ways can the integration of behavioral and social sciences approaches, such as formative and explanatory methods, improve the design and implementation of clinical trials for HBV cure research?
• How can understanding the lived experiences of HBV trial participants and their social support systems help identify key factors that influence intervention acceptability, adherence, and overall success and translation of cure interventions?
2. **Ethics**
• What ethical guidelines should be developed to address the challenges associated with HBV treatment discontinuations and finite treatments, including inclusion/exclusion criteria, monitoring strategies, and treatment restart criteria?
• What are ethical considerations for people with HBV and HIV in HBV cure research?
• How can informed consent be ensured in HBV cure trials, particularly regarding the use of the term “cure” and the understanding of the risks and benefits of treatment discontinuation?
• What are some of the modality or intervention-specific ethical considerations in HBV cure research?
• What are the ethical implications of involving marginalized populations (e.g., migrant populations, people who inject drugs) in HBV cure research, and how can their unique needs be addressed ethically?
• What ethical frameworks can be employed to ensure fair access to HBV cure research opportunities, especially for people in resource-limited settings and marginalized populations?
• How can HBV cure research minimize the risk of exacerbating stigma, particularly among people with both HIV and HBV, and how can social harm be prevented?
• How should trial designs for HBV cure research be ethically designed, particularly regarding the use of placebos and the inclusion of potentially vulnerable populations, such as children or those who are pregnant?
• What ethical considerations should be taken into account when developing interventions to mitigate third-party risks (e.g., HBV vaccination for partners) while respecting participant privacy?
• How can equity, human rights, and justice-informed paradigms guide the ethical conduct of HBV cure research to prevent the inadvertent creation of further health disparities or social harms?
3. **Community Engagement**
• How can community (and other stakeholder) groups (e.g., providers, researchers, and regulators) be engaged to understand their perspectives on HBV treatment discontinuations, particularly in the context of co-infections like HIV?
• What are the most effective methods for engaging people with lived experiences of HBV in the research process, and how can their voices be incorporated into trial design, recruitment, and decision-making?
• What strategies can be developed to engage underserved and marginalized communities in HBV cure research, particularly those in resource-limited settings or with limited English proficiency?
• How can we address the challenges posed by stigma in HBV cure research, especially for populations affected by dual stigma (e.g., people with both HBV and HIV)?
• What culturally appropriate communication strategies can be employed to ensure that affected communities understand complex HBV cure research terminology?
• How can community advisory boards be expanded or enhanced to better include voices from diverse regions and populations affected by HBV, particularly in underrepresented areas with high HBV prevalence?
• What are the best practices for ensuring that informed consent processes are culturally and linguistically appropriate for diverse patient populations, and how can these processes be improved for people with HBV?
• How can trust be built and maintained with communities throughout the HBV cure research process, from study design to dissemination of findings?
• What lessons can be learned from other fields, such as HIV cure research, to improve community engagement in HBV cure research, particularly in the development of models that promote equitable and effective participation?
4. **Translational and Implementation**
• How can the T0-T4 translational research framework be adapted and applied to HBV cure development to ensure the successful transition from basic research to real-world applications?
• What are the key factors influencing the scalability of HBV cure interventions across different healthcare systems, particularly in resource-limited settings?
• How can we develop cost-effectiveness models for HBV cure interventions to inform policy decisions and ensure equitable access, especially considering the high initial costs seen in hepatitis C cure implementation?
• What strategies can be implemented to ensure that HBV cure interventions are accessible to underserved populations, particularly in regions with high HBV prevalence and limited healthcare infrastructure?
• How can personalized care approaches be integrated into HBV cure therapies to optimize treatment for diverse patient populations, including those with co-infections or comorbidities?
• What are the essential components of monitoring and evaluation systems required to assess the long-term sustainability and effectiveness of HBV cures in real-world settings?
• How could lessons learned from the design, governance, and implementation of programs such as Gavi and PEPFAR be adapted to develop a coordinated global initiative for hepatitis B prevention, care and cure in highly endemic regions?
• How can healthcare systems effectively track and address barriers to the implementation of HBV cure interventions, especially in resource-limited settings with high co-infection rates (e.g., HBV and HIV)?
• What are the critical social, cultural, and political factors that need to be considered in the implementation of HBV cure interventions to ensure they align with local community needs and expectations?
• How can the lessons learned from hepatitis C cure implementation be applied to the global rollout of HBV cures to avoid disparities in access and cure outcomes?
• What role do capacity building efforts play in the successful adoption and implementation of HBV cure interventions worldwide, and how can these be effectively scaled?

Additionally, future research should consider the potential consequences of achieving HBV cure on other health outcomes. For example, efforts to identify and treat HBV may reveal undiagnosed hepatitis C cases, creating opportunities to expand HCV treatment and cure. Conversely, achieving HBV cure could unintentionally be associated with changes in sexual behaviors, such as potentially increasing other sexually transmitted infections, highlighting the need for integrated prevention strategies (112). Leveraging existing cohorts and infrastructure, fostering collaborations, and integrating lessons from related research fields will be crucial for implementing a multidisciplinary HBV cure research agenda that is ethically robust, socially acceptable, and sustainable.

## Conclusions

Advancing a robust, multidisciplinary HBV cure research agenda requires integrating acceptability, ethics, community, and equity considerations. Addressing psychosocial factors, treatment discontinuations, co-infections, and person-centered trial designs will help to ensure research is relevant and meaningful for diverse populations. Strategic collaborations, genuine stakeholder engagement, and investment in high-burden regions will be essential to translate scientific progress into lasting global health impact.

## Key References

- Adda G, Wang S. A Declaration from People Living with Hepatitis B: A Call for a Whole Person Approach. *Journal of Viral Hepatitis* 2023; 30(7): 603 (13).
  - The official declaration from people living with hepatitis B that calls for a whole-person approach to care and research.
- Amorosa VK, Aibana O, Shire NJ, Dorey-Stein Z, Ferrara T, Gilmore J, Kostman JT, Lo Re III, V. Willingness to Undergo a Repeat Liver Biopsy among HIV/Hepatitis C-coinfected and Hepatitis C Virus-monoinfected patients. Journal of Clinical Gastroenterology 2013; 47(5): 457 – 60 (53).
  - This survey study, conducted with 235 people with HCV in the context of care – not research, provides a rare account of the willingness to undergo liver biopsies. Perceived safety, the importance of the biopsy, and knowing someone who has undergone the procedure were positively associated with the willingness to undergo the biopsy.
- Borondy-Jenkins F, Ansah B, Chen J, Goldring A, Ibrahim Y, Issa S, Lesidrenska S, Machado T, Moore H, Njouom R, Okinedo P, Racho R, Scott L, Zovich B, Cohen C. Global Hepatitis B and D Community Advisory Board: Expectations, Challenges and Lessons Learned. Frontiers in Public Health 2024; 12: 1437502 (86).
  - Although not directly related to the field of HBV cure research, this paper presents findings from focus group discussions with 16 participants, who shared lessons learned from an inaugural global community advisory board focused on hepatitis B and delta clinical research.
- Cornberg M, Suk-Fong Lok A, Terrault NA, Zoulin F, and the 2019 EASL-AASLD HBV Treatment Endpoints Conference Faculty. Guidance for Design and Endpoints of Clinical Trials in Chronic Hepatitis B – Report from the 2019 EASL-AASLD HBV Treatment Endpoint Conference, Journal fo Hepatology 2020; 72: 539 – 57 (52).
  - This report provides guidance on the design and endpoints of clinical trials aimed at achieving a functional cure for HBV, based on discussions from a 2019 meeting of the European Association for the Study of the Liver (EASL) and the American Association for the Study of Liver Diseases (AASLD).
- Freeland C, Racho R, Kamishke M, Moraras K, Wang E, Cohen C. Cure Everyone and Vaccinate the Rest: The Patient Perspective on Future Hepatitis B Treatment. *Journal of Viral Hepatitis* 2021; 28: 1539 – 44 (32).
  - This qualitative interview study conducted 19 people with CHB in the Unted States found the majority expressed enthusiasm towards HBV functional cure but worried about potential side effects.
- Hardstock F, Sbarigia U, Kocaata K, Wilke T, Sylvester SV. Preferences of Patients with Chronic Hepatitis B – A Discrete Choice Experiment on the Acceptability of Functional Cure. *Patient Preference and Adherence* 2020; 14: 613 – 24 (31).
  - This discrete choice experiment conducted in Germany with 108 people with CHB found that efficacy, particularly sustained HBV remission, was the most important factor driving acceptability, followed by regimen type, safety, and physician visits, with participants preferring oral administration and fewer side effects, highlighting the need for future treatments to prioritize efficacy and convenience.
- Hendriks S, Pearson SD. Assessing Potentional Cures: Are These Distinctive Elements of Value Beyond Health Gain. *Journal of Comparative Effectiveness Research* 2021; 10(4): 255 – 65 (59).
  - This perspective manuscript, written outside the field of HBV cure research, argues that new elements of value unique to cures include freedom from the burden of ongoing treatment, the absence of a diseased identity, and a reduction in disease-related stigma – all of which can only be assessed through the thoughtful integration of socio-behavioral sciences.
- Lazarus JV, Ivancovsky Wajcman D, Pannain S, Brennan PN, Manolas MI, Jepsen P, Treloar C, Arora AK, Matthews PC, Picchio CA, White TM, Grebely J, Vaz J, Hagström H, Rabin KH, Isaacs S, Ribeiro RT, Roden M, Betel M, Willemse J, Díaz LA, Allen AM, Alkhouri N, Schattenberg JM, Mauricio D, Rinella ME, Pose E, Tsochatzis EA, Ninburg M, Cusi K, Alazawi W, Duseja A, Frühbeck G, Lofton H, Jaisinghani P, Kanwal F, Shiha G, Zelber-Sagi S, Holden L, Villota-Rivas M. The People-First Liver Charter. *Nature Medicine* 2025; 31(7): 2109 – 16.
  - The People-First Liver Charter advocates for the adoption of person-first language in liver disease care and research, emphasizing the need to prioritize the dignity and rights of individuals living with liver-related conditions.
- Mohtashemi N, Dubé K, Thio C, Song S, Patel S, Sugarman J, Bhattacharya D. Patient Acceptability of, and Attitudes Towards, Hepatitis B Cure Research – A Scoping Review and Identification of Knowledge Gaps. *Journal of Virus Eradication* 2023; 9: 100354 (30).
  - The scoping review on patient perspectives of hepatitis B functional cure research identifies two peer-reviewed articles (31,32) from studies conducted in the United States and Germany (high-income countries) and highlights gaps in understanding, emphasizing the need for further research.
- Revill PA, Chisari FV, Block JM, Dandri M, Gehring AJ, Guo H, Hu J, Kramvis A, Lampertico P, Janssen HLA, Levrero M, Li W, Liang TJ, Lim SG, Lu G, Capucine Penicaud M, Tavis JE, Thimme R, Members of the ICE-HBV Working Groups, ICV-HBV Stakeholders Chairs, ICE-HBV Senior Advisors, Zoulim F. A Global Scientific Stategy for Cure Hepatitis B. Lancet Gastroenterology and Hepatology 2019; 4: 545 – 58 (7).
  - This paper reports on two consultations involving 152 stakeholders from 21 countries, focusing on a global scientific strategy for an HBV cure.
- Richmond JA, Ellard J, Wallace J, Thorpe R, Higgs P, Hellard M, Thompson A. Achieving Hepatitis C Cure: A Qualitative Exploration of the Experiences and Meanings of Achieving Hepatitis C Cure using Direct Acting Antivirals in Australia. *Hepatology, Medicine and Policy* 2018: 3(8): 1 – 9 (34).
  - This qualitative interview study provides a rare account of the experience of being cured of hepatitis. Twenty participants described a range of benefits from achieving HCV cure, including improved psychological well-being, reduced anxiety about developing liver disease or cancer, and a diminished fear of transmitting the virus.
- Sugarman J, Revill P, Zoulim F, Yazdanpanah Y, Janssen HLA, Lim, SG, Lewin SR. Ethics and Hepatitis Cure Research. *Gut* 2017; 66(3): 389 – 92 (6).
  - This seminal review on the ethics of HBV cure research focuses on five key areas: 1) risks of interventions, 2) outcome measures, monitoring, and modeling, 3) participant selection, 4) language and informed consent, and 5) fairness.
- Sugarman J. Ethics of HIV and Hepatitis B Cure Research. *Current Opinion in HIV/*AIDS 2020; 15(3): 180 – 4 (27).
  - This comparative review of HBV and HIV cure research ethics highlighted under-addressed areas in HBV cure research, including stakeholder engagement, obligations to third parties, stigma assessment, and the need for a deeper understanding of the attitudes, beliefs, and experiences of HBV cure research participants.
- Wallace J, Richmond J, Howell J, Hajarizadeh B, Power J, Treloar C, Revill PA, Cowie B, Wang S, Stoové M, Pedrana A, Hellard M. Exploring the Public Health and Social Implications of Future Curative Hepatitis B Interventions. *Viruses* 2022; 14 (2542): 1 – 14 (101).
  - This original research article presents findings from 31 stakeholder interviews conducted in Australia, exploring the public health and social implications of future HBV cure interventions. The discussion focuses on five key topics relevant to community engagement and implementation research: how HBV “cure” is framed, health system implementation, clinical infrastructure, equity and access, and the broader social implications of an HBV cure.

## Data Availability

No datasets were generated or analyzed for this manuscript.
